# Laboratory evaluation of oral fluid for syphilis screening among clinic users from Lima, Peru

**DOI:** 10.1128/spectrum.01298-24

**Published:** 2024-12-10

**Authors:** Jazmin Qquellon, Silver K. Vargas, Francesca Vasquez, E. Michael Reyes-Diaz, Kelika A. Konda, Carlos F. Caceres, Jeffrey D. Klausner

**Affiliations:** 1 Centro de Investigación Interdisciplinaria en Sexualidad, SIDA y Sociedad, Universidad Peruana Cayetano Heredia, Lima, Peru; 2 Department of Population and Public Health Sciences, Keck School of Medicine, University of Southern California, Los Angeles, California, USA; Icahn School of Medicine at Mount Sinai, New York, New York, USA

**Keywords:** oral fluid, syphilis screening, rapid diagnostic tests, Peru

## Abstract

Serological tests for syphilis require equipment unavailable in many health centers; however, point-of-care testing facilitates rapid screening using finger-prick whole blood samples. A further improvement could be oral fluid for ease of use. We evaluated the performance of treponemal antibody point-of-care testing for syphilis screening using oral fluid samples. We recruited users of STI clinics in Lima, Peru. We collected oral fluid using the OraSure collection device (OraSure Technologies Inc., USA) according to the manufacturer’s instructions and serum from each participant. Oral fluid and serum were analyzed using the SD Bioline Syphilis 3.0 rapid test (Standard Diagnostics Inc., Korea). Serum was also tested using RPR (RPR slide test, Wiener Laboratorios SAIC, Argentina) and TPPA (Serodia, Fujirebio Diagnostics Inc., Japan). We assessed oral fluid rapid test overall percent agreement, sensitivity, specificity, and Kappa coefficient against serum SD Bioline Syphilis, TPPA, and RPR. Among 323 participants, nearly half (51.3%) reported prior syphilis. The overall percent agreement, sensitivity, specificity, and Kappa coefficient of oral fluid were 71.0% (95% CI: 65.6%–75.8%), 78.1% (71.1%–83.7%), 63.5% (55.8%–70.6%), and 0.42% (0.32%–0.52%), respectively, versus serum SD Bioline Syphilis. A similar performance was obtained versus serum TPPA. When limiting the sample to TPPA-reactive with RPR titer ≥1:8, the sensitivity increased to 88.1% (75.0%–94.8%), whereas the specificity did not vary (65.3% [57.2%–72.6%]). We observed a good performance of the rapid treponemal test using oral fluid. Further investigations are needed to improve the specificity of oral fluid as a potential sample for accurate syphilis screening.

**IMPORTANCE:**

Point-of-care tests are essential for rapid and efficient screening of syphilis, especially in low-resource settings. Oral fluid specimens offer a noninvasive, accessible, and practical alternative to traditional blood samples. Our study evaluated the performance of a rapid treponemal test using oral fluid specimens. We found that the rapid treponemal test using oral fluid had good sensitivity compared with the rapid treponemal test using serum and the non-treponemal RPR test. This suggests that oral fluid could be a viable option for syphilis screening, facilitating broader and faster access to diagnosis and treatment. However, further studies are needed to improve the specificity of this method to ensure accurate screening in diverse clinical scenarios.

## INTRODUCTION

Syphilis is a curable bacterial sexually transmitted infection (STI) caused by the spirochete *Treponema pallidum* (TP). The global burden of syphilis was 7.1 million new cases in 2020 (1), disproportionately affecting key populations such as men who have sex with men (MSM), transgender women (TW), and female sex workers (FSW) (2). Recently, a systematic review and meta-analysis estimated the global prevalence of syphilis in the last two decades and found a prevalence of 7.1% among MSM and 16.6% among other populations, including male sex workers, TW, and TW sex workers (3). Regarding FSW, the median syphilis seroprevalence reported by 38 countries as part of the Global AIDS Monitoring was 3.2% (4). In addition, there was a concern that almost half of the countries in Latin America and the Caribbean have reported an increasing trend of syphilis cases among pregnant women between 2015 to 2020 (5). In Peru, a study using treponemal rapid tests estimated that the lifetime prevalence of syphilis was 37.7% among MSM, 54.8% among TW, and 3.4% among FSW (6). Also, the incidence of active syphilis was 1.02 per 1,000 pregnant women screened between 2011 and 2017 (7).

Serological syphilis diagnosis involves two types of tests, treponemal and lipoidal antigen (non-treponemal), which are performed independently but combined in diagnostic algorithms to identify infected individuals (8, 9). Treponemal antibodies remain present even if the individuals received proper and timely treatment, whereas non-treponemal antibodies vary depending on the treatment response. However, lipoidal antigens can cross-react with other clinical conditions, resulting in biological false positives (10, 11). In addition to the testing targets used, conventional laboratory tests have some limitations, such as requiring specific equipment for proper processing and trained personnel for performing and interpreting results (12). In recent years, point-of-care (POC) tests for syphilis have been successfully developed for the detection of treponemal antibodies, being useful for screening in high-prevalence populations or among pregnant women, where vertical transmission can be prevented. One key advantage of POC tests is the possibility of performing the tests and treatment on the same day, using a variety of sample types including finger prick capillary blood, venous blood, plasma, and serum (13). The collection of other types of specimens, such as urine, dried blood spots, saliva, or oral fluids, could also be beneficial for testing.

Prevention strategies have introduced less invasive sampling techniques as an alternative to the traditional diagnosis of HIV and tuberculosis (14, 15), making plausible the search for other types of samples for syphilis testing, such as oral fluid. In addition, a previous study found that treponemal antibodies in oral fluid specimens can be detected using immunofluorescent assays (16). More recently, a study evaluated if treponemal rapid tests could work if used with oral fluid and found good sensitivity of this assay when compared with confirmatory treponemal tests in serum (17). However, more data regarding the use of oral fluid for syphilis testing are needed. Evaluating the performance of these testing alternatives in laboratory and clinic-based settings is a high priority. Therefore, we aim to evaluate the performance of oral fluid for syphilis testing compared with standard syphilis testing using serum from STI clinic users.

## RESULTS

Among 323 individuals enrolled in this study, the median age was 31 years (interquartile range [IQR]: 26–38), and 71.5% were male (see Table 1). Most of them (60.4%, 195/323) were MSM, 26 (8.0%) were TW, 85 (26.3%) were sex workers (83 female sex workers and two male sex workers), and 17 (5.3%) were general population. Clinical and serological examination found that 39 (11.1%) participants had active syphilis infection; of these, three were diagnosed as primary syphilis cases, four as early latent syphilis cases, and 32 as late latent syphilis cases. Almost a third of participants, 100 (31.0%), were living with HIV, and 165 (51.1%) reported a prior syphilis infection, of whom 72 (43.6%) were diagnosed in the last year. Laboratory results indicated that 177 (54.8%) individuals had a positive TPPA result, 114 (35.3%) had a reactive RPR result (72 with an RPR titer 1:1 to 1:4 and 42 with an RPR titer ≥1:8), and 164 (50.8%) had a positive serum SD Bioline Syphilis result.

**TABLE 1:** Characteristic of STI clinic users in Lima, Peru, 2022

Variables			N (%)
Age (years)^*a*^			31 (26–38)
Sex			
	Male		231 (71.5)
	Female		92 (28.5)
Type of population			
	MSM		195 (60.4)
	TW		26 (8.0)
	Sex workers		85 (26.3)
	General population		17 (5.3)
Living with HIV			
	No		223 (69.0)
	Yes		100 (31.0)
Prior syphilis infection			
	No		153 (47.4)
	Doesn´t know		5 (1.5)
	Yes		
		Only once	102 (31.6)
		More than once	63 (19.5)
Time of last prior syphilis infection			
	≤ 6 months ago		23 (13.9)
	6–12 months ago		49 (29.7)
	>12 - ≤ 60 months ago		63 (38.2)
	> 60 months ago		30 (18.2)
Active syphilis infection			
	No		284 (87.9)
	Yes (stage)		
		Primary	3 (1.0)
		Early latent	4 (1.2)
		Late latent	32 (9.9)

MSM: Men who have sex with men, TW: Transgender Women.

^*a*^ Median (p25-p75).

The SD Bioline Syphilis using oral fluid was compared with treponemal serologic tests such as serum SD Bioline Syphilis and TPPA (see Table 2). When comparing the SD Bioline Syphilis oral fluid to serum, the overall percent agreement, sensitivity, specificity, and Kappa coefficient of SD Bioline Syphilis using oral fluid was 71.0% (95% CI: 65.6%–75.8%), 78.1% (95% CI: 71.1%–83.7%), 63.5% (95% CI: 55.8%–70.6%), and 0.42 (95% CI: 0.32–0.52), respectively. We found similar results when we used the serum TPPA as a comparator, with a slight increase in specificity (65.1%, 95% CI: 57.0%–72.3%).

**TABLE 2:** Performance of the use of oral fluid in the treponemal rapid test versus treponemal serologic comparators

(1)	Serum SD bioline syphilis rapid test		Total	Sensitivity (95% CI)	Specificity (95% CI)	Overall percent agreement (95% CI)	Kappa coefficient (95% CI)
	Positive	Negative					
Oral fluid rapid test positive	128	58	186	78.1% (71.1%–83.7%)	63.5% (55.8%–70.6%)	71.0% (65.6%–75.8%)	0.42 (0.32–0.52)
Oral fluid rapid test negative	36	101	137				
Total	164	159	323				

(2)	Serum TPPA		Total	Sensitivity (95% CI)	Specificity (95% CI)	Overall percent agreement (95% CI)	Kappa coefficient (95% CI)
	Positive	Negative					
Oral fluid rapid test positive	135	51	186	76.3% (69.5%–81.9%)	65.1% (57.0%– 72.3%)	71.2% (65.9%–72.3%)	0.42 (0.32–0.52)
Oral fluid rapid test negative	42	95	137				
Total	177	146	323				

**Fig 1:**
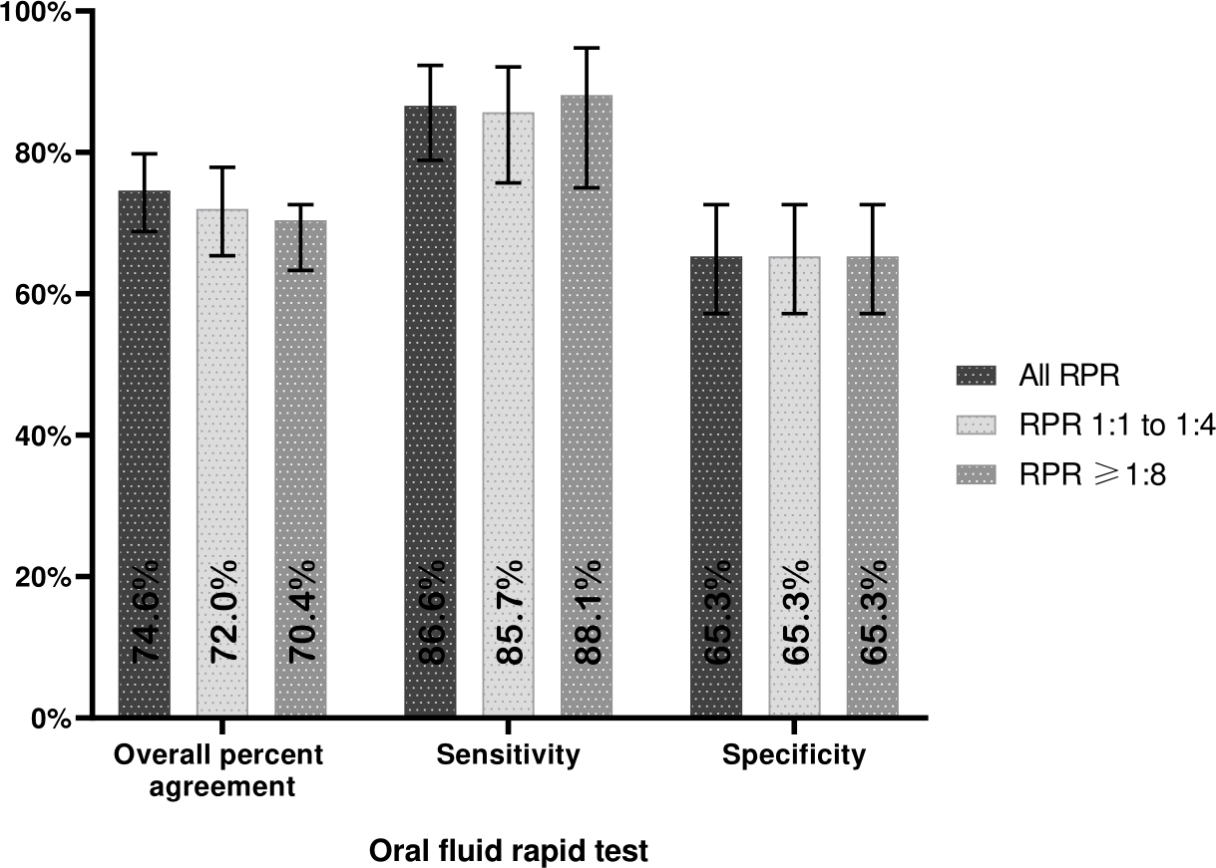
Performance of the use of oral fluid in the treponemal rapid test versus non-treponemal serologic comparators. All RPR results (*N* = 256) were categorized as non-reactive (*N* = 144), RPR titer 1:1–1:4 (*N* = 70), and RPR titer ≥1:8 (*N* = 42). Kappa coefficient of oral fluid versus all RPR = 0.50 (0.40–0.60), RPR 1:1–1:4 = 0.45 (0.33–0.56), and RPR ≥1:8 = 0.39 (0.27–0.50). Samples with a positive RPR result were also positive for TPPA, and samples with a negative RPR result were also negative for TPPA.

The performance of SD Bioline Syphilis using oral fluid against different non-treponemal serologic comparators yielded an increase in the overall percentage of agreement and sensitivity when we used all RPR results as a comparator (74.6% [95% CI: 68.8%–79.8%] and 86.6% [95% CI: 78.9%–92.3%], respectively) (see Fig. 1). Similar results were maintained when using only RPR titer 1:1–1:4 as a comparator (72.0% [95% CI: 65.4%–77.9%] and 85.7% [95% CI: 75.7%–92.1%], respectively) and when using only RPR titer ≥1:8 as a comparator (70.4% [95% CI: 63.3%–76.9%] and 88.1% [95% CI: 75.0%–94.8%], respectively). The specificity using RPR non-reactive as a comparator was 65.3% (95% CI: 57.2%–72.6%). The Kappa coefficient of oral fluid decreased with increasing RPR titer when compared with all RPR results, the kappa was 0.50 (95% CI: 0.40–0.60), using only RPR titer 1:1–1:4, it was 0.45 (95% CI: 0.33–0.56), and using only RPR titer ≥1:8, it was 0.39 (95% CI: 0.27–0.50).

Among people living with HIV, we selected those with an RPR reactive and TPPA positive or RPR non-reactive and TPPA negative (*n* = 73). The performance of their oral fluids using a rapid treponemal test showed a high overall percentage of agreement and sensitivity when we used serum rapid test as a comparator (82.2% [95% CI: 71.5%–90.2%] and 90.6% [95% CI: 79.3%–96.9%], respectively) and when we used serum TPPA as a comparator (84.9% [95% CI: 74.6%–92.2%] and 92.5% [95% CI: 81.8%–97.9%], respectively). In addition, we found an increase in the Kappa coefficient of oral fluid when we compared it with serum rapid test: 0.53 (95% CI: 0.31–0.75) and when we compared it with serum TPPA: 0.60 (95% CI: 0.40–0.80). However, we did not find an improvement in the specificity of oral fluid using treponemal serologic tests: 60.0% (95% CI: 36.1%–80.9%) compared with serum rapid test and 65.0% (95% CI: 40.8%–84.6%) compared with serum TPPA.

Regarding anti-TP ELISAs, we tested 111 paired serum and oral fluids for IgG/IgM screening (48/144 both TPPA and RPR non-reactive, 23/65 TPPA reactive and RPR non-reactive, 24/70 TPPA reactive and RPR ≤1:4, and 16/72 TPPA reactive and RPR ≥1:8) and 107 paired specimens for IgG-only screening (48/144 both TPPA and RPR non-reactive, 21/65 TPPA reactive and RPR non-reactive, 24/70 TPPA reactive and RPR ≤1:4, and 14/72 TPPA reactive and RPR ≥1:8). We found that 59 (53.2%) had a positive serum IgG/IgM result, whereas 50 (46.7%) had a positive serum IgG-only result. The overall percent agreement, sensitivity, and specificity of anti-TP Elisa for IgG/IgM screening using oral fluid was 53.2% (95% CI: 43.4%–62.7%), 11.9% (95% CI: 5.9%–22.5%), and 100.0% (95% CI: 93.1%–100.0%), respectively, when using anti-TP Elisa for IgG/IgM screening with serum as the comparator. We found similar results when we compared the anti-TP for IgG-only screening using oral fluid against the same assay method using serums (55.1% [95% CI: 45.2%–64.8%], 4.0% [95% CI: 1.1%–13.5%], and 100.0% [95% CI: 93.7%–100.0%], respectively).

## DISCUSSION

In this study, we assessed the performance of a commercial rapid test for syphilis screening using oral fluid as a specimen. Our findings showed that SD Bioline Syphilis using oral fluid had a good overall percentage agreement, sensitivity, and specificity against treponemal and non-treponemal comparators. Compared with treponemal serologic tests (rapid test and TPPA), the overall percentage agreement was up to 71.2%, the sensitivity up to 78.1%, and the specificity up to 65.1%. However, the overall percentage agreement and sensitivity increased when compared with samples with equal serum TPPA and RPR results, and when we used TPPA positive and only RPR titer ≥1:8.

In our study, we found higher sensitivities among those with RPR titers ≥ 1:8 (88.1%) and among those living with HIV (92.5%). As described by Bristow et al., the sensitivity of the rapid test using oral fluid improves with increasing RPR titer (17). In addition, the synergistic interaction between syphilis and HIV increases infectivity and delays the clearance of *T. pallidum* (18). This suggests that the SD Bioline Syphilis test using oral fluid can be valuable for syphilis screening, especially in high-risk populations.

Previously, the oral fluid was described as a useful sample where DNA, RNA, and proteins circulate (19). In addition, Wang et al. detected TP DNA in saliva at all stages of syphilis, considering this sample appropriate for molecular diagnostics, especially using nested polymerase chain reaction (20). However, some difficulties have been observed in the use of rapid immunoassays, affecting its sensitivity, due to the lower concentration of IgG compared with those found in serum samples (21). In addition, studies have described factors associated with false negatives in HIV rapid tests performed with oral fluid. In a study, failure to follow all steps and errors during the testing stage were the most common for self-testers (22). In clinical research studies, factors significantly associated with false negatives were test operator, clinical site, use of PrEP, low plasma viral load, and time to kit expiration (23).

The specificities were relatively low, around 65%, indicating that almost 35% of the negative samples yielded false positive results in the rapid test using oral fluid, likely due to the cross-reactivity with antibodies to non-pathogenic treponemas in oral fluid, such as *Treponema denticola* (24). In Peru, a study reported a prevalence of >75% among patients with periodontal disease and approximately 60% in healthy individuals (25). Given the high prevalence of *T. denticola*, one potential solution to mitigate cross-reactivity would be to pre-treat the oral fluid sample. This is a procedure already implemented in tests such as Fluorescent Treponemal Antibody-Absorption (FTA-ABS) and TPPA, where the dilution buffer contains *T. denticola* antigens to adsorb antibodies that could otherwise lead to cross-reactivity. We encourage future studies to search for other reasons for low specificities using oral fluid and to apply new methods for their improvement.

Regarding the anti-TP ELISAs, we found poor performance using oral fluid. This aligns with a previous study that reported low sensitivity of TPPA when using oral fluid (26). We suspected that the lower concentrations of antibodies in oral fluid, compared with serums, may have reduced the sensitivity of anti-TP ELISA tests. In addition, oral fluid extracted by centrifugation after freezing and thawing could have reduced IgM and IgG levels. In contrast, a time-resolved fluorescence immunoassay for detecting treponemal IgG in oral fluid samples achieved high sensitivities, from 87.5% for primary syphilis to 100% for secondary syphilis. In that study, oral fluid was pretreated using a transport medium (including gentamicin and amphotericin B) and filtered through a 0.2 µm filter (27). Other studies have reported good performances of oral fluid antibody detection immunoassays for the diagnosis of HIV (sensitivity ≥96% and specificity ≥97%) and hepatitis A, B (HBs Ag), and C (sensitivity/specificity of ≥92%/≥86%, ≥85%/≥94%, and ≥84%/≥99%, respectively) (28, 29). Although the use of oral fluid as a diagnostic specimen has been increasing, the lack of standardization methods for the collection, pretreatment, processing, and storage of oral samples remains a challenge for scale-up (30).

Oral fluid tests are non-invasive, require minimal training for sampling, and are often preferred for patients. Despite this, we acknowledge that oral fluid collection and processing involve multiple steps, which may be considered a limitation, especially in comparison to POC syphilis testing using capillary blood obtained by fingerstick. However, previous studies that evaluate the performance of the SD Bioline Syphilis test using finger-prick blood have reported sensitivities between 47% and 96%, with the highest sensitivities from studies involving exclusively people living with HIV (31–33). Therefore, we consider that the SD Bioline Syphilis test using oral fluid could be comparable with the sensitivity of the SD Bioline Syphilis test using capillary blood.

POC tests have the potential to significantly enhance and broaden the scope of screening initiatives when effectively integrated into control programs (34). Screening for syphilis using a rapid treponemal test is a strategy that, when complemented with non-treponemal tests such as RPR, can help differentiate active and past infections, thereby reducing persistent positive results, particularly in high-prevalence populations (35). The incorporation of treponemal tests using oral fluid into the syphilis diagnostic algorithm could also benefit lower-prevalence populations, including female sex workers and the general population who access STI clinics. Furthermore, integrating dual treponemal/nontreponemal tests into this algorithm could enhance diagnostic accuracy while minimizing unnecessary treatment. Future studies should consider evaluating the performance of these dual tests in comparison to standard platforms, as they may offer improved diagnostic coverage and practical advantages by utilizing accessible specimens like oral fluid, particularly in resource-limited settings.

Our study had the following limitations: the pre-processing of oral fluid samples was performed according to the Oral Specimen Collection Device specifications, including sample collection and storage. Samples were collected and stored at the STI clinics, then transported to the evaluation laboratory, where the sample was extracted from the pad by centrifugation and then frozen until processing. This procedure and storage may have affected the quality of oral fluid, decreasing the concentration of antibodies to levels insufficient to react with antigens coated on the membrane of the rapid test. In addition, we did not assess the concentration of anti-TP antibodies. Moreover, the rapid test we used for syphilis testing was only intended for use with serum, plasma, or whole blood. Despite this, our study included a large number of people, including vulnerable populations, and was able to demonstrate to STI clinic attendees the easy noninvasive collection of oral fluid for syphilis screening.

In conclusion, we describe the good performance of oral fluid in a treponemal rapid test. Oral fluid could be an alternative to the common samples used for syphilis screening by rapid tests. However, further research is still needed to improve specificity, perhaps by adding a sample pre-treatment step to eliminate potential interferences or by using tests that detect multiple specific anti-*Treponema pallidum* antibodies. We also recommend implementing field testing to avoid freezing and transport of collected samples.

## MATERIALS AND METHODS

### Specimen collection

Individuals who routinely attended STI clinics in Lima, Peru, in 2022 were recruited by a group of healthcare providers trained in the management of STI and HIV among the general population, MSM, TW, and FSW. Participants met the following criteria: 18 years old or older, able to give informed consent, and willing to provide oral fluid specimens and venipuncture whole blood specimens. At the clinic sites, syphilis screening was performed using a treponemal antibody rapid test (Alere Determine™ Syphilis TP, Alere Inc., USA) and the non-treponemal Rapid Plasma Reagin (RPR) test (RPR slide test, Wiener lab, Argentina). In addition, participants completed a 15-min questionnaire while they were awaiting routine STI test results. They reported general demographic characteristics, sexual identity, history of syphilis, presence of genital lesions at enrolment, recent antibiotic use, HIV status, and among those living with HIV, their engagement in HIV care.

We collected oral fluids using the Orasure Oral Specimen Collection Device (Orasure Technologies, Inc., USA). Participants placed the collection pad between the cheek and gums until it was moist, approximately 3 min to allow the pad to absorb as much oral fluid as possible, and then placed it inside the Orasure transport tube device with preservative buffer. Additionally, a blood sample was collected by venipuncture into serum gel tubes and centrifuged for 10 min at 2,500 rpm. The serum was separated and aliquoted into microtubes (1 mL each). Both specimens were properly stored at –20°C until transportation to the Sexual Health Laboratory at Universidad Peruana Cayetano Heredia. Once arrived, we followed the laboratory procedures recommended by the manufacturer to recover the oral fluid from the transport device (36). The oral fluid transport device was thawed and then inverted (tip facing upwards and pad facing the cap) to break the tip against the inner wall of the centrifuge tube. It was centrifuged at 3,000 rpm for 15 min; the oral fluid specimen was extracted and stored at −20°C until processing.

### Laboratory diagnosis

Oral fluid and serum were paired and analyzed using the treponemal SD Bioline Syphilis 3.0 rapid test (Standard Diagnostics Inc., Korea). This immunochromatographic assay contains a membrane coated with recombinant TP15 and TP17 antigens used for the capture and detection of specific treponemal antibodies of all isotypes (IgG, IgM, and IgA). Oral fluid was processed using 30 µL + 2 drops of sample diluent buffer, whereas serum was processed according to the manufacturer’s instructions (10 µL + 4 drops of sample diluent buffer). Before evaluating the SD Bioline test using oral fluid, we verified its performance using previously characterized positive and negative control serum samples. The control line was visible and consistently appeared in all tests, regardless of syphilis status. Serum was also tested using the Treponema Pallidum Particle Agglutination (TPPA) test (Serodia, Fujirebio Diagnostics Inc., Japan).

Additionally, we randomly selected paired serum and oral fluid specimens and tested using two enzyme immunoassays for the semi-quantitative determination of IgG/IgM and IgG-only antibodies to TP (Anti-TP ELISA for IgG/IgM screening, EUROIMMUN Inc., Germany and Anti-TP ELISA for IgG screening, EUROIMMUN Inc., Germany, respectively). The microplate wells of both Anti-TP ELISA tests were coated with TP15, TP17, TP47, and TmpA recombinant antigens. Oral fluid was processed without sample dilution (100 µL was added directly to the well plate), whereas serum was processed with a dilution of 1:101 (according to the manufacturer’s instructions). For each sample, the ratio value was calculated (ratio: extinction of patient sample over the extinction of the calibrator 2). A ratio ≥1.1 was interpreted as a positive result, ≥0.8 to <1.1 as a borderline result, and <0.8 as a negative result.

### Statistical analysis

The performance of the rapid treponemal test using oral fluid was assessed against the following comparators: (i) serum SD Bioline Syphilis (ii), serum TPPA (iii), All RPR results (iv), only RPR titer 1:1 to 1:4, and (v) only RPR titer ≥1:8. For non-treponemal results, positive RPRs were also positive for TPPA, and negative RPRs were also negative for TPPA. We also evaluated the oral fluid performance in only people living with HIV using treponemal serologic tests as comparators. The performance of anti-TP ELISAs using oral fluid was compared with their performance using serum. The sensitivity and specificity were estimated using the exact binomial method to determine 95% confidence intervals (*CIs*). The overall agreement was determined by dividing the number of agreement scores by the total count of scores and the Kappa values were calculated using Cohen’s Kappa statistic. Data analysis was performed using the Stata statistical software (version 17.0, Stata Corporation, USA), whereas the figure was performed using GraphPad Prism software (version 8.0, GraphPad Software, Inc., USA).
